# Equity and coverage of insecticide-treated bed nets in an area of intense transmission of *Plasmodium falciparum *in Tanzania

**DOI:** 10.1186/1475-2875-8-65

**Published:** 2009-04-16

**Authors:** Jubilate Bernard, George Mtove, Renata Mandike, Frank Mtei, Caroline Maxwell, Hugh Reyburn

**Affiliations:** 1 National Malaria Control Programme, Ministry of Health and Social Welfare, United Republic of Tanzania, Dar es Salaam, Tanzania; 2 National Institute for Medical Research in Tanzania, Amani Centre, Tanga, Tanzania; 3 Kilimanjaro Christian Medical Centre, Moshi, Tanzania; 4 London School of Hygiene and Tropical Medicine, Keppel St, London, WCIE 7HT, UK

## Abstract

**Background:**

There is no clear consensus on the most sustainable and effective distribution strategy for insecticide treated bed nets (ITNs). Tanzania has been a leader in social marketing but it is still not clear if this can result in high and equitable levels of coverage.

**Methods:**

A cluster-randomized survey of ITN and bed net ownership and use was conducted in a rural area exposed to intense *Plasmodium falciparum *transmission in NE Tanzania where ITN distribution had been subject to routine delivery of national strategies and episodic free distribution through local clinics. Data were collected on household assets to assess equity of ITN coverage and a rapid diagnostic test for malaria (RDT) was performed in all ages.

**Results:**

Among 598 households in four villages the use of any or insecticidal bed nets in children less than five years of age was 71% and 54% respectively. However there was a 19.8% increase in the number of bed nets per person (p < 0.001) and a 13.4% increase in the number of insecticidal nets per person (p < 0.001) for each quintile increase in household asset score. The odds of being RDT-positive were reduced by more than half in the least poor compared to the poorest households (OR 0.49, 95% CI 0.35–0.70). Poorer households had paid less for their nets and acquired them more recently, particularly from non-commercial sources, and bed nets in the least poor households were less likely to be insecticidal compared to nets in the poorest households (OR 0.44, 95% CI 0.26–0.74).

**Conclusion:**

Marked inequity persists with the poorest households still experiencing the highest risk of malaria and the lowest ITN coverage. Abolition of this inequity within the foreseeable future is likely to require mass or targeted free distribution, but risks damaging what is otherwise an effective commercial market.

## Background

It was been estimated that 63% all childhood deaths would be averted if existing and affordable interventions were effectively delivered [1]. Among these, the use of insecticide treated bed nets (ITNs) in young children has the potential to reduce all-cause mortality in children under the age of five years by 17% in malaria endemic areas[2] but of 34 African countries reporting to RBM in 2005 only one had achieved the target of 60% coverage of ITNs in under-five children (revised in 2005 to 80%) [3].

The reasons for such disappointing ITN coverage over more than 15 years since the first studies showing their potential to reduce mortality are diverse but centre on the failure to agree and implement a distribution strategy that can achieve high coverage in an equitable and sustainable way [4-6]. A variety of strategies have been used to boost coverage including the promotion of the commercial market [7]. enhancing the commercial sector through a variety of promotions and subsidies (social marketing) [8,9] and, more recently, the provision of free ITNs to vulnerable groups through community distributions or primary care clinics [10-12]. The trend over this period has been for increased levels of subsidy and the debate has more recently focused on free vs subsidized distribution, the key issues being equity and coverage (favouring free distribution) and sustainability (favouring social marketing) [13].

Tanzania is one of the countries that has made a strong commitment to socially marketed ITNs; in 1998 the first ITN effectiveness trial demonstrated a reduction in child mortality associated with socially marketed ITNs in southern Tanzania [8] and a national programme of socially marketed nets was instigated in 1998 as a collaboration between Population Services International and the Tanzanian National Malaria Control Programme (NMCP)[14]. From 2004 the Tanzanian Net Voucher Scheme (TNVS) was introduced to improve equity of ITN coverage among the poorest communities while preserving the commercial sector; this provides pregnant mothers with a voucher that can be used to purchase an ITN of their choice in a commercial outlet for less than $1, the balance being redeemed by the shopkeeper. Against this background free ITN distribution occurs sporadically in Tanzania funded by a variety of NGOs or other charities.

Many of the studies assessing impact of different ITN strategies have compared specific ITN delivery campaigns with baseline coverage. However, the reality for most rural communities in malaria-endemic areas is that ITNs are offered through various sources, each associated with differences in cost and insecticide treatment. Thus net coverage at any point in time is the result of a variety of initiatives that have been operational during the lifetime of currently owned nets.

This study aimed to identify how different ITN strategies have resulted in equity and coverage of ITNs in a rural, malaria-endemic area that, with the exception of a few well-defined research sites that were excluded from the study, has not been subject to intense local ITN campaigns.

## Methods

### Study area

The study was conducted in Muheza District near the coast of NE Tanzania, a site of malaria research for a number of years and where *P. falciparum *transmission is intense and perennial with seasonal peaks. [15] The local economy based on subsistence farming and commercial fruit growing. Child mortality is typical of that in Tanzania generally at 176/1000 children in the first 5 years of life [16].

### Sampling and study procedures

The survey lasted between April and May 2008 to coincide with the rainy season and thus peak seasonal malaria transmission. In order to ensure that villages were typical of the high malaria transmission known to occur in the area we identified 72 villages that had each contributed more than 10 admissions for severe malaria to the local district hospital where such admissions were documented for 1 year in 2006–7. The district town was excluded as were villages that were known to have been the site of ITN trials where nets had been freely distributed. From the 68 eligible villages, four were randomly sampled and from each of these villages 10 'balozis' (a Tanzanian 10-house unit for which a register is kept in the village office) were randomly sampled and all houses within the selected balozis were visited for the survey. With adjustment for clustering the sample size was calculated to estimate a 20% prevalence of ITN ownership +/-5% with 95% confidence.

The responsible adult present in the house was interviewed regarding the number of nets in the house, their source, price (converted to U.S. dollars using the exchange rate at the time of the survey) and whether or not they were treated with insecticide in the previous 6 months or were long-acting insecticidal nets. Nets were inspected wherever possible but evidence of whether they were insecticidal was taken from the respondent only. Data were also collected on house construction and household possessions to compile an aggregated score of socio-economic status. Data on individual use of a bed net the previous night was collected and a finger-prick blood sample was obtained from all available household members for a malaria rapid diagnostic test (RDT) using Paracheck™, an HRP-2 based test that has compared well with expert slide reading [17].

Data were double entered in MS Access (MS Corp, Redmond, Va) and analysed using Stata 10 (Statacorp, College Rd, Tx). A socio-economic score was derived using principal component analysis of household assets as described by Filmer and Pritchet (2001) [18]. The first principal component accounted for 40.3% of the observed variation in household assets and, using this component, quintiles of a weighted score were derived from the 6 most correlated assets (non-mud floor, non-grass roof, radio, bicycle, mobile phone and other than locally made oil burner as a light source).

### Ethics

The study was approved by the ethical review boards of the Tanzanian National Institute for Medical Research and LSHTM. Written consent was obtained from all study subjects or their parent/guardian if under 15 years of age.

## Results

A total of 604 households in four villages (range 120–212 households per village) were included in the study and six were excluded from the analysis due to incomplete data on household possessions. In the resulting 598 households there were 1,911 individuals reported to be resident, the median household size was 4 and mean (median) age was 23.7(15) years, 42% of households included a child under the age of five years and 1,136(61%) of subjects were female, as were 76% of those reporting for the household.

### Bed net ownership by household asset score

**Figure 1:**
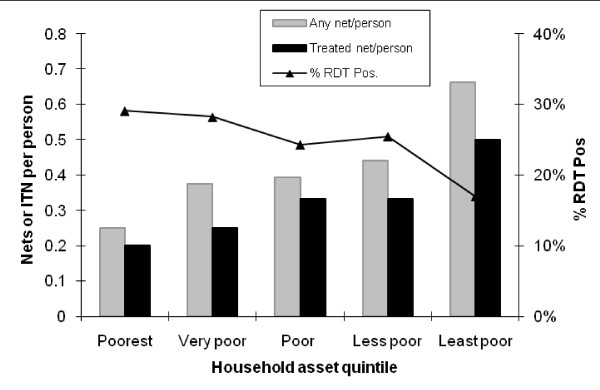
**Mean number of bed nets per household member and proportion who were RDT-positive by household asset quintile**.

Household asset scores are shown in Table 1. In addition to individual variation the mean household asset score varied by village (range 2.6 to 3.7). Overall, 401(67%) households had any net and the mean number of any nets and insecticidal nets per household member varied by household asset score (Figure 1). The ratio of household ownership of any net between the poorest and least poor was 0.58. Using regression analysis with adjustment for clustering between balozis, there was a 19.8% increase in the number of bed nets per person for each unit increase in household asset quintile (p < 0.001) and a 13.4% increase in the number of insecticidal nets per person (p < 0.001).

**Table 1:** Household asset quintiles in 598 households surveyed.

**Asset Quintile**	**Households (%)**	**Non-mud floor**	**Non-grass roof**	**Bicycle**	**Mobile phone**	**Radio**	Non-koroboi light*
Poorest	110 (18.3%)	0%	0%	0%	0%	0%	0%
Very poor	130 (21.7%)	0%	38%	0%	0%	62%	0%
Poor	115 (19.2%)	3%	38%	40%	23%	70%	10%
Less poor	121 (20.2%)	35%	71%	55%	45%	79%	18%
Least poor	122 (20.4%)	89%	89%	73%	70%	93%	70%

*Locally made oil-burning light source, non-koroboi assumes kerosene or electric light source.

**Figure 2:**
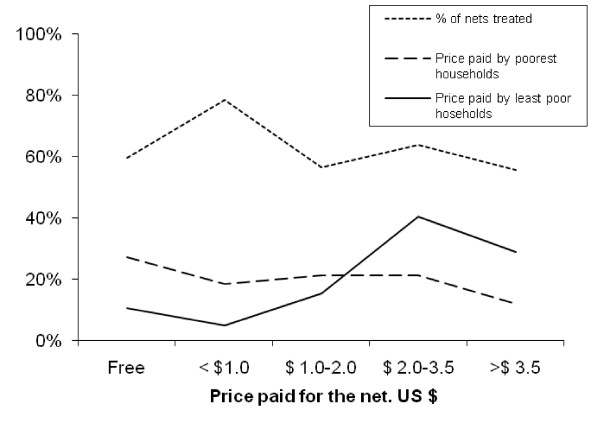
**Reported price paid for bed nets and the proportion of nets that were treated by poorest and least poor households**.

**Figure 3:**
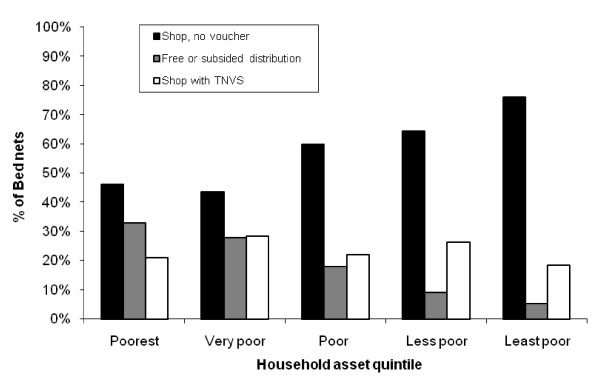
**Reported source of bed nets by household asset quintile**.

The mean price of nets acquired within the last year was $1.9 while for nets that were 3 or more years old it was $2.6 and for the poorest households these prices were $1.2 and $2.4 respectively (Figure 2). The source of bed nets varied by household asset quintile, with nets that had been distributed through a non-commercial 'malaria campaign' making up a third of nets in the poorest households but only 5% of nets in the least poor households (Figure 3).

Just over half of the nets (335/570, 59%) that had been purchased from a shop were insecticidal and for nets that had been bought using a TNVS voucher this fell to 125/250(50%). However, 165/214 (77%) of nets distributed through a 'malaria campaign', whether subsidized or free, were insecticidal. Nets used by adults (over 15 years of age) were less likely to be insecticidal than those used by children under the age of 15 years (OR 0.66, p = 0.012) although this did not vary between younger (less than five years) and older (5–15 years) children (OR 0.98, p = 0.95).

Factors associated with whether nets had been treated with insecticide were assessed using a logistic regression model; nets that were insecticidal were more likely than non-insecticidal nets to have been donated or obtained at a subsidized price, to have been acquired more recently and a higher proportion of nets in the poorest households had been treated with insecticide (Table 2).

**Table 2:** Logistic regression model of factors associated with whether nets were insecticidal(= 1) or not insecticidal (= 0)

	**OR**	**P**	**95% CI**
Poorest	1		
Very poor	1.22	0.38	0.78–1.90
Poor	0.78	0.22	0.52–1.16
Less poor	0.47	< 0.001	0.31–0.72
Least poor	0.44	0.002	0.26–0.74
Net age < 1 yrs	1		
Net age 1–2 yrs	0.58	0.014	0.38–0.90
Net age 2–4 yrs	0.50	0.006	0.31–0.82
Net age > 4 yrs	0.30	< 0.001	0.18–0.50
Shop-purchased, no voucher	1		
Donated net	1.54	0.003	1.04–2.23
TNVS Purchase	0.53	< 0.001	0.38–0.73

The reported travelling time to the nearest shop selling bed nets was relatively short; 60% of households were within a 20 minute journey and 82% were within a one-hour journey of a shop selling nets and net ownership of nets did not vary systematically by travel time.

#### Individual bed net use

**Figure 4:**
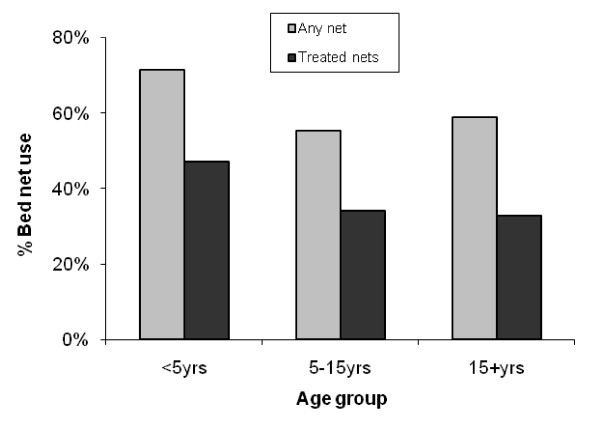
**Use of any bed net and insectide-treated net the previous night by age**.

Among children under the age of five, 215/301(71%) were reported to have slept under a bed net in the previous night and 159(54%) under an insecticidal net. Use of any net or insecticidal nets was more common under five years of age compared to older children and adults (71% compared to 57%, p < 0.001 and 47% compared to 33%, p < 0.001 respectively) (Figure 4).

In addition there were gender differences in net use; among children under the age of five years more boys than girls were reported to have slept under a bed net the previous night (115/148, 78% compared to 100/153, 65%, p = 0.018) although this difference was not observed for ITNs (54% compared to 52%, p = 0.78). Over the age of 15 years more women than men reported sleeping under a bed net (63% compared to 49%, p < 0.001) and the same difference was observed for insecticidal nets (35% compared to 30%, p = 0.05).

#### *P. falciparum *exposure, age and bed net use

The proportion of subjects positive for RDT varied by household asset quintile (Figure 1) but also by age, reported use of a bed net and balozi of residence. A logistic regression model was used that included RDT result (positive/negative) as the dependent outcome and household asset score, use of bed nets and age as independent variables with adjustment for clustering on balozi of residence. Independently of age, village of residence and use of bed nets, a positive RDT result was associated with low household asset score (Table 3).

**Table 3:** Logistic regression model of RDT result (positive = 1, negative = 0), household asset index, age and use of bed nets after adjustment for village clustering.

	**OR**	**p**	**95% CI**
Poorest	1		
Very poor	0.9	0.236	0.76–1.07
Poor	0.74	< 0.001	0.64–0.86
Less poor	0.82	0.048	0.67–0.99
Least poor	0.48	0.002	0.29–0.76
No use of any bed net	1		
Use of any bed net	0.82	0.404	0.52–1.13
No use of an ITN	1		
Use of insecticidal net	1.2	0.562	0.70–1.91
Age < 5 yrs	1		
Age 5–15 yrs	1.42	0.210	0.82–2.48
Age 15+ yrs	0.29	< 0.001	0.14–0.57
Male	0.94	0.219	0.79–1.05

## Discussion

The study area is similar to many rural, malaria-endemic settings in Africa that have been subject to a variety of distribution strategies in recent years. With the exception of a few villages that had been the site of research (and which were excluded from this study) the area has not been the focus of exceptional efforts to boost bed net coverage but had benefited from national social marketing strategies that had operated in the last few years supplemented by episodic, small-scale free or heavily subsidized net distribution from various charities operating from local clinics. Nets in the latter category are difficult to quantify due to their episodic nature. Until recently they were discouraged nationally and no further detail is available. However, our data suggest that such donations have been particularly successful in targeting the poorest households in recent years.

### Equity of ITN coverage

The findings of this study, like those of Schellenberg *et al *[19], reveal that while rural African villages often have an appearance of uniform poverty there are definite socioeconomic differences that are reflected in marked differences in the use of health interventions. As so often with health risks and provision of services, the equity in coverage of ITNs was double-edged; the poorest households had the lowest level of ITN possession and, independent of bed net use, were at greatest risk of current or recent *P. falciparum *infection. True equity (equality of access according to need) would actually require greater coverage of ITNs among the poorest households compared to the least poor, the opposite to the difference that we observed.

The socio-economic differential in coverage was less marked for ITNs compared to untreated nets and this was associated with recently acquired ITNs and non-commercial distribution (free or highly subsidized). The TNVS is likely to have contributed to improved bed net coverage although over half of the nets acquired under the scheme were not impregnated. This is disappointing as, although the voucher can be used to buy any net, basic nets in Tanzania are 'bundled' with insecticide from the manufacturer and raise the possibility of interference in the packaging. There were also anecdotal reports that shopkeepers, when presented with vouchers instead of cash, tended to increase the base price of a net and it seems likely that the poorest households are particularly vulnerable to these sort of sharp practices as they are less mobile and may be less aware of their rights.

There was also evidence of inequity based on gender; while net coverage of young boys was only slightly higher than girls, the difference was statistically significant and reflects similar differences that have been observed in children with severe malaria [20].

### Insecticide treatment

Overall almost half of the nets in the study were not impregnated. While this may represent an improvement over past rates it still suggests a failure to realise much of the individual benefit of sleeping under a bed net [21]. There is well-documented evidence of the mass effect of ITNs [22] and high levels of ITN coverage have recently been associated with marked reductions in the burden of malaria [23,24]. In this study, non-impregnated nets were associated with paradoxically lower risk of RDT-positivity compared to impregnated nets although the effect was not significant. It seems likely that this was the result of residual confounding not controlled for by our asset score and suggests that the excess risk of malaria in the poorest households may be even higher than documented by the study.

Thus the case for free insecticide treatment as a social good seems to be particularly strong. While future net sales are likely to be restricted to long-acting insecticidal nets, re-impregnation of the huge numbers of existing untreated nets is unlikely to be achieved through social marketing; Guyatt *et al *[25] found that in Kenya, while high rates of insecticide treatment could be achieved through providing free insecticide, the rates declined dramatically when charges were introduced.

### Free or social marketed ITNs?

Tanzania has pioneered initiatives in socially marketed nets and our study provides, albeit incomplete, evidence on its effect where most circumstances favour its success. Our results demonstrate significant progress since 2005 when a survey in the study area found that bed net use in young children was only 22%, only 10% of which had been treated with insecticide [26]. Many of these gains must be credited to social marketing.

In an rural area of Tanzania where social marketing had been rigorously promoted, Nathan et al [27] documented that the proportion of the poorest households with a bed net increased from less than a quarter in 1997 to more than a half in 2000 and an increase the ratio of 'any net in the household' between the poorest and least poor from 0.3 to 0.6. Coverage in our study area five years later showed a strikingly similar pattern and suggests a lag of several years between areas with intense promotion and those subject to the consequent national roll-out that in our study area has been supplemented by non-commercial distribution that accounted for a third of ITNs in the poorest households.

These results suggest that, in spite of significant gains, social marketing as a single strategy is insufficient to meet current targets or to further reduce the inequity in ITN coverage amongst the poorest communities [28]. In Tanzania mass free ITN distribution is planned starting in 2009 but the effect of sustained free distribution on the existing commercial outlets and reliance on the continued donor support both remain uncertain [29].

## Conclusion

In spite of significant gains in bed net coverage achieved by social marketing there is still marked inequity in coverage of bed nets among the poorest and most at-risk households. Recent free or heavily subsidized distributions have resulted in reducing inequity in coverage of insecticidal nets but abolition of social inequity in ITN ownership in poor communities is likely to require mass or targeted free distribution. However, the long-term effect on the commercial market in ITNs remains uncertain.

## Competing interests

The authors declare that they have no competing interests.

## Authors' contributions

JB designed the study, was involved in collection of data and participated in data analysis and writing the manuscript. GM contributed to the study design, data analysis and made critical comments on the manuscript. RM contributed to the write up and made critical comments on the manuscript. FM was responsible for data management, contributed to the analysis and made critical comments on the manuscript. CM was responsible for leading the data collection and made critical comments on the manuscript. HR was involved in all stages, coordinated the analysis and drafted the manuscript.
